# Attempting to synthesize lasso peptides using high pressure

**DOI:** 10.1371/journal.pone.0234901

**Published:** 2020-06-24

**Authors:** Mateusz Waliczek, Magdalena Wierzbicka, Maciej Arkuszewski, Monika Kijewska, Łukasz Jaremko, Priyadharshni Rajagopal, Kacper Szczepski, Amanda Sroczyńska, Mariusz Jaremko, Piotr Stefanowicz

**Affiliations:** 1 Faculty of Chemistry, University of Wrocław, Wroclaw, Poland; 2 Biological and Environmental Sciences & Engineering Division (BESE), King Abdullah University of Science and Technology (KAUST), Thuwal, Saudi Arabia; Rijksuniversiteit Groningen, NETHERLANDS

## Abstract

Lasso peptides are unique in that the tail of the lasso peptide threads through its macrolactam ring. The unusual structure and biological activity of lasso peptides have generated increased interest from the scientific community in recent years. Because of this, many new types of lasso peptides have been discovered. These peptides can be synthesized by microorganisms efficiently, and yet, their chemical assembly is challenging. Herein, we investigated the possibility of high pressure inducing the cyclization of linear precursors of lasso peptides. Unlike other molecules like rotaxanes which mechanically interlock at high pressure, the threaded lasso peptides did not form, even at pressures the high pressure up to 14 000 kbar.

## Introduction

Lasso peptides belong to a specific class of natural peptides characterized by a unique “knot” structure motif [1]. These compounds are synthesized ribosomally and modified post-translationally. Thus, the peptide precursor is genetically encoded, but the target structure is formed by a set of several enzymes. These peptides are built from a macrolactam ring formed from an isopeptide bond between an N-terminal amino acid residue (usually glycine or alanine) and a side chain of aspartic or glutamic acid. The remaining C-terminal chain is threaded through the macrolactam ring and resembles a lariat knot, which can be divided into a loop and a tail [2]. The unique topology of lasso peptides is sustained by steric interactions provided by the presence of bulky amino acid residues (e.g. tryptophan in the exocyclic part of the peptide). The broad interest in lasso peptides is due to both their extraordinary topology and their biological activity [3, 4, 5, and 6]. For example, a recent biological study of lassomycin showed some activity against *Mycobacterium tuberculosis* [7]. However, its structure remains uncertain, and data reported in the literature show conflicting information about whether or not lassomycin has the characteristic “knot” motif [7, 8, and 9]. This does not imply, however, that the structures of lasso peptides are completely undeterminable. There are many examples of lasso peptides for which the topology has been established with certainty, including sungsanpin [10] and chaxapeptin [11]. Sungsanpin is a 15-amino acid peptide isolated from a marine-derived microorganism (*Streptomyces)* discovered in Korea. Additionally, Um and Co. reported that sungsanpin inhibits activity the human lung cell line A549. The sequence of the second mentioned biomolecule is largely similar to the first, (11/15 aa), despite differences in the C-terminal sequence. Sterically hindered amino acids in the tail of lasso peptides prevent the lasso peptide from the transitioning to the unthreaded form. Since the discovery of the first lasso peptide in 1991, the total number of lasso peptides studied and reported in the literature has increased, particularly in recent years, which brings the total number of discovered and researched lasso peptides to >40 [12,13,14]. Our opinion is that the chemical synthesis of lasso peptides would open new doors to designing new analogues and to studying their biological activities. According to data reported in the literature, all attempts to synthesize natural lasso peptides have been unsuccessful, due to the lack of a specific “knot” structure [9]. Those attempts produced branched-cyclic peptides in an unthreaded form. Recently, Gomez and Co. [15, 16] proposed a new method of chemical synthesis in which the sequences of lasso peptide precursors are modified to allow the formation of an additional intramolecular bond (disulfide or lactone one). In this method, the author assumes that the formation of the lactam bond in the cyclic peptide is followed by a reduction of the disulfide bridge, or that hydrolysis of the ester bond may provide a natural, threaded form of the lasso peptide. The current study, however, had mostly negative results: no lasso peptide formed; rather, unthreaded structures of regular branched-cyclic peptides were produced. Recently, a cryptand-imidazolium complex was designed, and applied to the chemical synthesis of the lasso peptide. However, the proposed method is not generalized, and requires an individualized design cryptand and for each lasso peptides [17].

Although the chemical synthesis of lasso peptides presents many challenges, many examples of the successful chemical synthesis of rotaxanes [18, 19, 20, 21, 22] including peptide-based rotaxanes [23, 24] have been reported in the literature. These molecules show a topology similar to that of a lasso peptide, as the systems are considered to be mechanically interlocked molecular architectures. In the case of [2] rotaxanes, the chain, threaded through the macrocyclic ring, is a separate molecule that is not connected to the ring. Data reported in the literature show that the synthesis of rotaxanes is definitely more efficient under elevated pressure [25, 26]. This may be the result of temperature influencing the kinetics and thermodynamics of rotaxane formation. Since these systems are structural analogues, similar effects may be also expected for lasso peptides.

**Fig 1:**
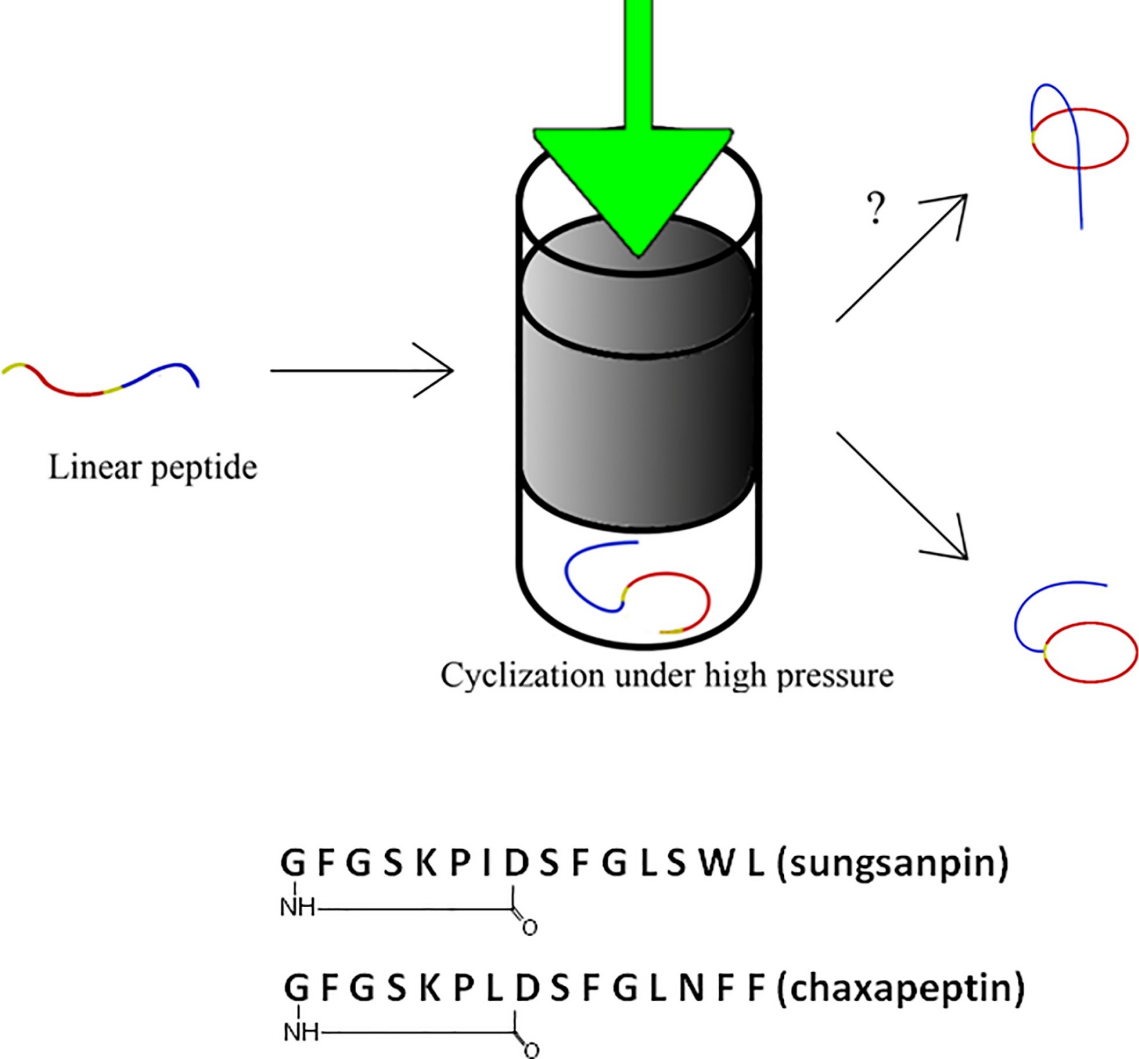
Scheme representing our approach applied in the synthesis of lasso peptides and the sequences of sungsanpin and chaxapeptin.

This high-pressure approach, however, has not been tested for lasso peptide synthesis. Therefore, this paper is the first attempt to investigate the combined effects of high pressure and solvent on the cyclization of peptides in order to obtain lasso peptides, especially for the cyclization of the linear core peptide (LCP) of lasso peptides. Herein, we investigated the effect of high pressure in an attempt to synthesize lasso peptides. We have attempted to synthesize the natural lasso peptides sungsanpin, chaxapeptin, and their respective analogues. We assumed that performing the cyclization step under elevated pressure would result in the formation of a more compact molecule, corresponding to the threaded form of a lasso peptide, characterized by a lower specific volume; therefore, we expected that the folding process would be more efficient at high pressure (Fig 1). To the best of our knowledge, there are no data available in the literature regarding this kind of experiment.

The pressure influences reaction equilibria according to Le Chatelier-Brown's principle. If the reaction volume (ΔV_rxn_) is > 0, applying pressure shifts the equilibrium towards the products. The difference of Vs for individual molecules (isolated molecules in gas phase) is definitely lower for native lasso peptide, which is documented by ion mobility mass spectrometry [15]. The experimental data indicate that the collisional cross section for the lasso form is lower than that of the regular, unthreaded peptide. However, solvation may change the Vs relationships between the threaded and unthreaded peptide. Predicting this effect is difficult, since it may depend on the applied solvent. In our manuscript, we present our experimental approach to solve this problem. We did this by performing cyclization of lasso peptides LCPs under varying conditions including several different solvents and pressures.

Using high pressure may create beneficial conditions for folding the LCP to the native conformation of the lasso peptide. Data reported in the literature show that high pressure facilitates the formation of rotaxanes [21, 22]. Numerous data confirm that protein conformation is sensitive to high-pressure [27, 28, 29, 30]. It has been demonstrated that pressure applied in the range 1–8 kBar influences protein structure, including ubiquitin or lysozyme, which are known for their stability [31]. For the high-pressure cyclization of LCPs of lasso peptides, several methods were used:

1. The cyclization of the LCP of a lasso peptide immobilized on a solid support: This method is based on the solid-phase peptide synthesis (SPPS), which was previously described [32]. An LCP of sungsanpin and chaxapeptin is synthesized, and then subjected to on-resin cyclization using several solvents and elevated pressure during the coupling.
2. The use of a sungsanpin analogue in solution with the C-terminal amide and lysine protected by a benzyloxycarbonyl group. In this case, the cyclization is performed under high pressure (13000 Bar).
3. High-pressure cyclization based on native chemical ligation. This procedure was previously used for homodetic and heterodetic peptides in an aqueous solution [33].
4. Gomez`s method combined with high pressure [15].

The obtained products were monitored by LC-MS, using a SIM (selected ion monitoring) mode. According to literature data, the threaded and unthreaded forms of a lasso peptide exhibit different chromatographic behaviours, which usually results in a clear separation of the two forms (threaded and unthreaded) [34,35,36,37].

## Results and discussion

### Cyclization of the linear core peptides of sungsanpin and chaxapeptin on solid support

The purpose of this study was to test different reaction conditions in order to assess the possible synthesis of lasso peptides. To do so, we synthesized a LCP of sungsanpin and chaxapeptin on both the Chemmatrix Amide Resin and Chemmatrix Wang Rink. Choosing this type of resin was dictated by the possibility of application of many solvent characterized by differential polarity. For the synthesis, we used Fmoc-Asp (O-2-PhiPr)-OH because of its lability of the side chain protection, thereby allowing the cyclization of the Asp side chain carboxyl group with the α-amine of the peptide. We did not expect that assembling these peptides in such manner would enable the formation of the lasso topology, as Lear and Co. [9] reported that the chemical synthesis of lassomycin produced a branched-cyclic peptide. Thus, the regular solid-phase peptide synthesis was ineffective in forming a threaded structure. However, we performed the on-resin cyclization in order to obtain a reference branched-cyclic peptide. The structure of the purified product was confirmed by NMR analysis, specifically by 2D homonuclear TOCSY and ROESY experiments.

**Fig 2:**
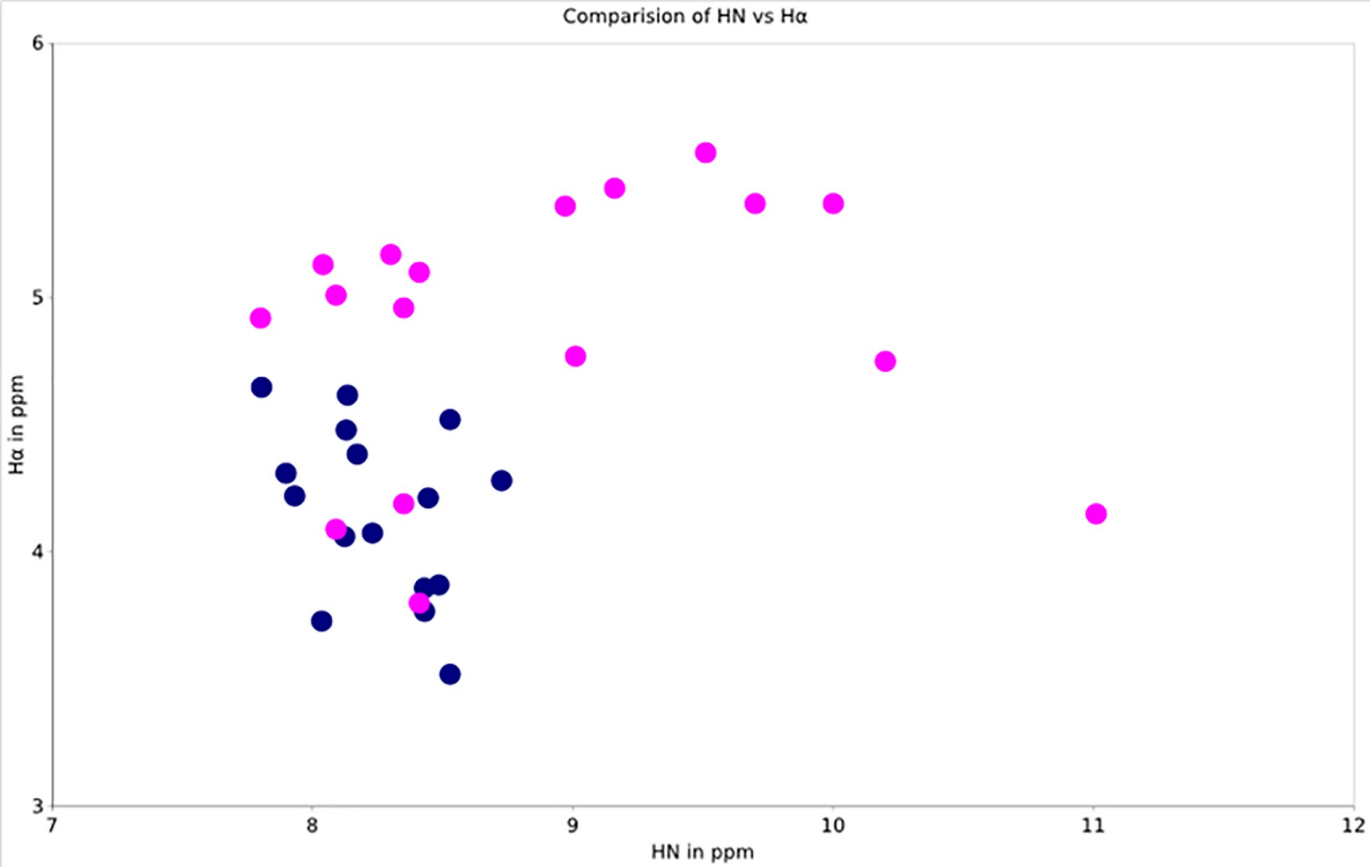
The graph resembling the 2D DQF-COSY spectra for the synthetic sungsanpin (I) branched-cyclic peptide in H_2_O/D_2_O (90/10% v/v) (Blue) and natural sungsanpin in pyridine-d_5_ (Pink) (Um et al., 2013 [10]) with the correlation of Hα and HN signals (in ppm) on the OY and OX axes, respectively.

As one can see from Fig 2, the sungsanpin in our conditions does not adopt a “lasso” conformation in the water solution after it dissolves. The signal dispersion is reduced as compared to the lasso form observed for the peptide in pyridine-d_5_. Analysis of Cα chemical shift values assigned to sungsanpin (S1 Table of S1 Data), reveals in water, that they are mostly elevated in comparison to those assigned in pyridine [10]. This suggests that the peptide most likely adopts a partially helical conformation in aqueous solution.

**Fig 3:**
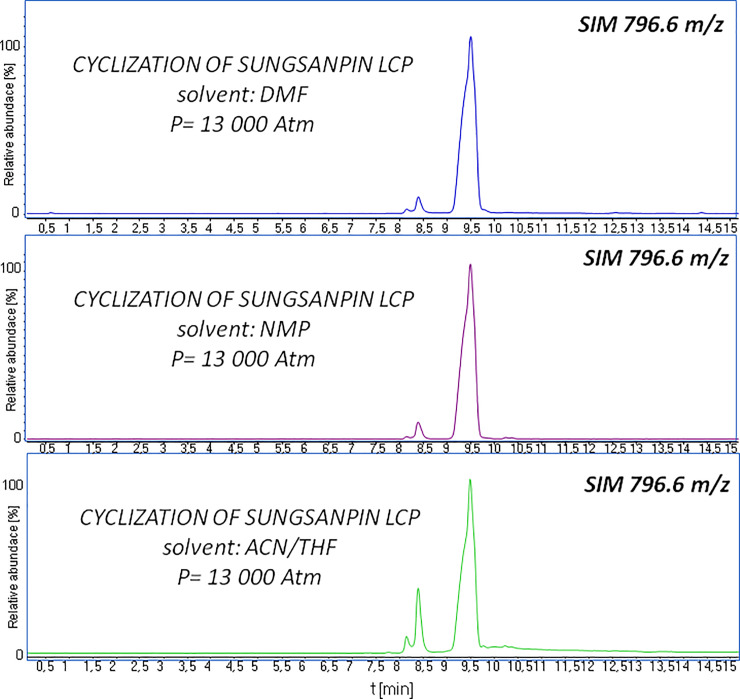
LC-MS chromatogram after cyclization of the sungsanpin LCP analogue acquired in SIM mode (m/z 796.6) under high-pressure, and using several solvent systems.

Our main goal was to study how high pressure influences the assembly of peptides exhibiting a characteristic knot structure. In our experiment, we applied a pressure of 13 000 bar, the highest pressure available on the piston apparatus. We tested the high-pressure on-resin cyclization of sungsanpin-NH_2_ and that of chaxapeptin-NH_2_ LCP analogues, using the following set of solvents: DMF, NMP, and THF/ACN. We chose polar and non-polar solvents to enforce conformational changes. As mentioned in the introduction, a lasso peptide differs from its branched-cyclic peptide analogue in terms of its chromatographic behaviour [34]. Therefore, we analyzed the reaction mixtures by LC-MS, using a selected ion monitoring mode (SIM). The results of the cyclization of the LCP of sungsanpin performed under high-pressure conditions are presented in Fig 3. In this figure, we note the presence of an additional signal (R_f_8.5), whose intensity increased when the peptide was cyclized in a THF/ACN mixture. In this case, we found the surface area ratio between these two peaks to be 20:80.

**Fig 4:**
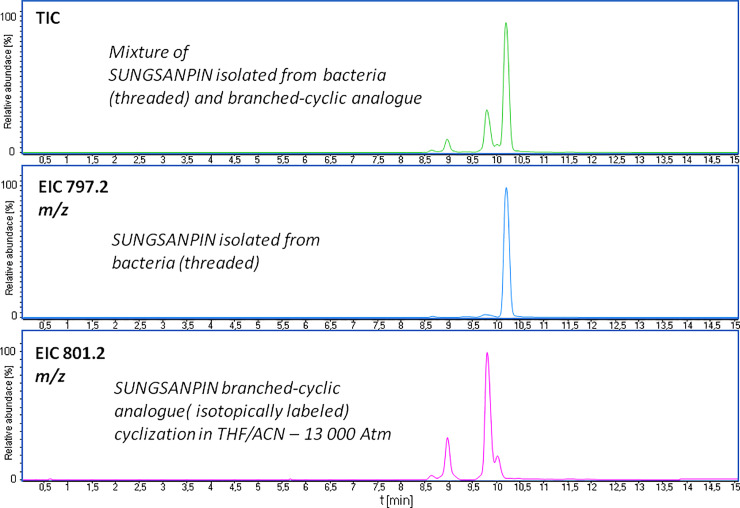
LC-MS-SIM chromatogram obtained for the mixture of natural sungsanpin (threaded) and its synthetic isotopically labelled branched-cyclic analogue: A. Total Ion Current (TIC) for the mixture of peptides; B. Extracted Ion Chromatogram (XIC) for natural sungsanpin; C. XIC for isotopically labelled synthetic sungsanpin branched-cyclic analogue obtained after a high-pressure cyclization.

Moreover, the LC-MS/MS analysis showed a similar fragmentation pattern, although the MS/MS spectrum of the signal at 8.5 min was characterized by lower intensities for most peaks. Additionally, we confirmed the identity of these two signals, using a high-resolution mass spectrometer because the triple quadrupole mass analyser has insufficient mass accuracy. So far, the obtained results did not show any evident formation of the specific lasso topology. One potential method to be explored, in order to achieve this goal, is NMR, which we also discussed in this section. However, we decided to compare our results with chromatographic data of the natural sungsanpin. For this reason, we synthesized the isotopically labelled LCP of sungsanpin (see S3 Fig of S1 Data), using a Chemmatrix Wang resin, which, in turn, allowed the assembly of the original α-carboxyl group-containing analogue. During the SPPS synthesis, we applied Fmoc-Lys-OH-^13^C_6_,^15^N_2_. The isotopologues have the same retention time as revealed by LC-MS analysis, but they have different masses. This makes it easy to differentiate from non-labelled compounds. The obtained LCP was subjected to a high-pressure cyclization, in a THF/ACN solvent system. The synthetic, isotopically labelled sample and the natural sungsanpin were pooled and analyzed by LC-MS. If the expected lasso form appeared, it would be distinguished from the bacterial form by the *m/z* ratio. Results are presented in Fig 4. The analysis of XIC for both peptides revealed differences in retention times, and showed a longer t_R_ for the threaded form. In our case, the additional signal formed after high-pressure cyclization exhibited a shorter t_R_ than that of the branched-cyclic peptide, which clearly excluded the possible formation of a lasso peptide.

The obtained data prompted us to answer questions regarding the peptide structure that was simultaneously formed with a branched-cyclic peptide as a result of high-pressure coupling and that exhibited the same molecular mass. In our opinion, there are two possible explanations: 1) dehydration of the linear peptide, either in solution or gas phase, possibly from a serine residue, for example. However, in the case of a gas-phase water loss, the dehydrated and the linear peptides should have the same retention times. But this was not consistent with our results. Furthermore, when taking into account the assumption that a serine is dehydrated, we should expect an increase in retention, due to the increased hydrophobicity of the product; 2) the formation of an aspartimide.

**Fig 5:**
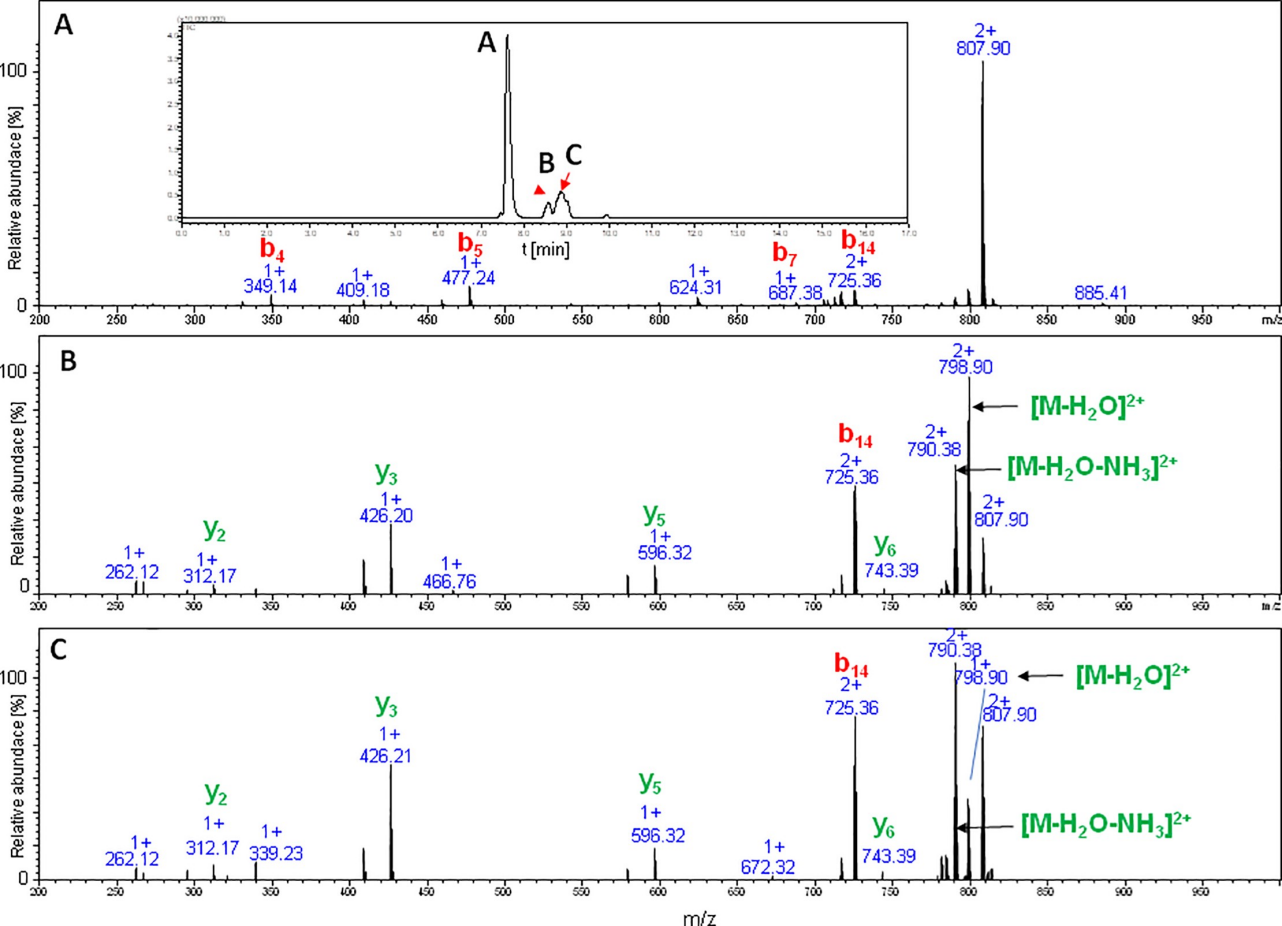
LC-MS/MS spectrum obtained for cyclization of the chaxapeptin-amide LCP analogue under high pressure in a mixture of ACN/THF. Fragmentation spectra of the branched-cyclic peptide (B, C) and the additional signal (A) are shown in the figure.

This modification leads to the formation of an isobaric compound with a branched-cyclic peptide (likewise with a lasso peptide), as well as a preservation of the linear nature of peptide. Thus, the formation of an aspartimide may explain the shorter retention time of the additional signal, in comparison with the cyclic product. This assumption was confirmed by the MS/MS spectrum of the products formed during the high pressure cyclization of the LCP of chaxapeptin (Fig 5).

### Solution-phase cyclization of the linear core peptides of sungsanpin and chaxapeptin

**Fig 6:**
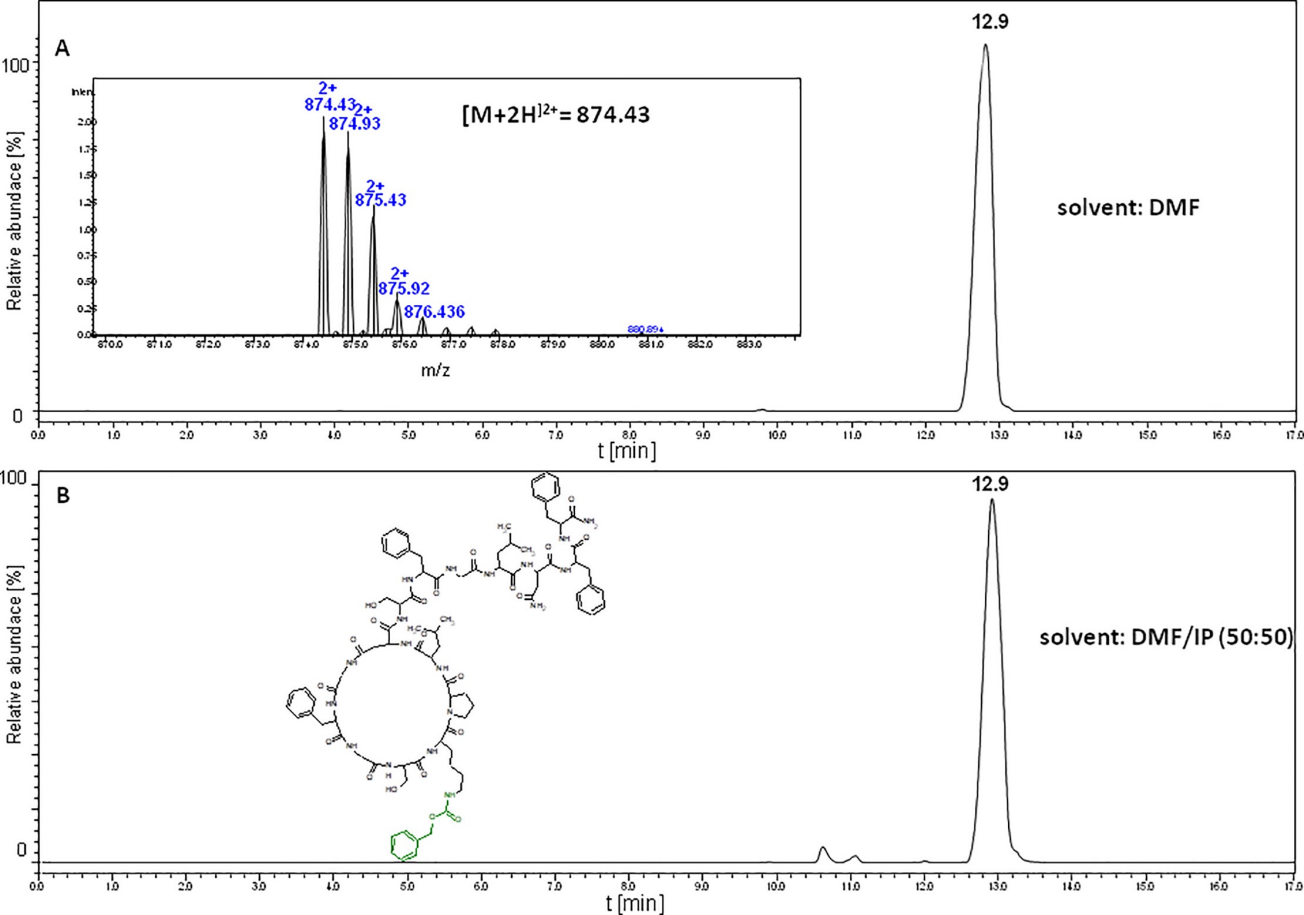
LC-MS chromatogram (XIC) obtained for the cyclization of the chaxapeptin-amide LCP analogue, containing the benzyloxycarbonyl protection group (Nε-Z-Lys): A. cyclization in DMF under high pressure; B. cyclization in the mixture of DMF/IP under high pressure.

**Fig 7:**
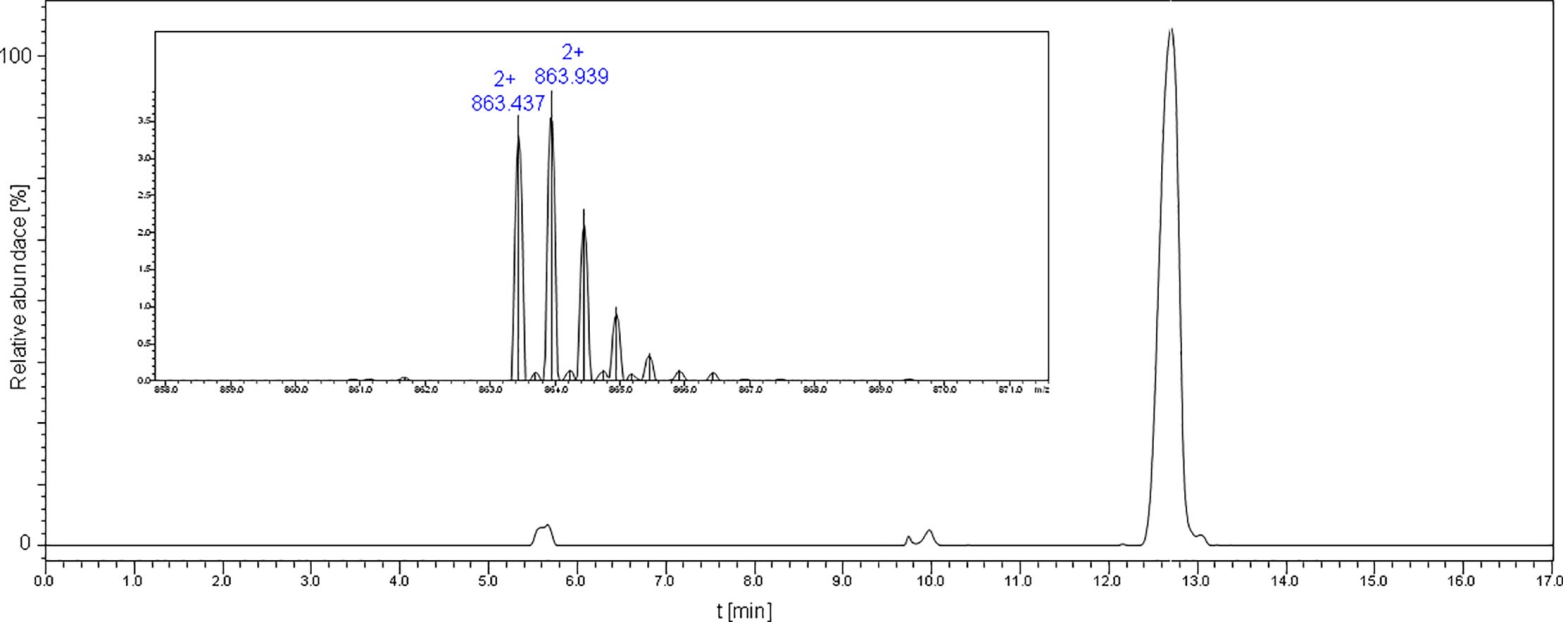
LC-MS chromatogram (XIC) obtained for cyclization of the sungsanpin-amide LCP analogue, containing benzyloxycarbonyl protection group (Nε-Z-Lys)—cyclization in DMF under high pressure in the mixture of DMF/IP (50:50).

The next step was to study the cyclization of the LCPs of sungsanpin and chaxapeptin in solution. There is a big difference between these two approaches, since the peptide is not attached to the resin after elimination of the protection groups, with the exception of benzyloxycarbonyl (Z) protection group of the lysine side chain. This protection was introduced during the SPPS synthesis by design because of its stability in standard cleavage conditions (TFA) so that no risk of undesirable coupling of the aspartic acid side chain with the ε-amino group of lysine could occur. Samples of LCP of sungsanpin and chaxapeptin were subjected to coupling using PyAOP (2-fold excess) and several solvent systems. Similar to the previous section, the reactions were carried out both in atmospheric and high pressure conditions on the piston apparatus. Thus, the aim of these experiments was to examine the possibility of a chemical synthesis of peptides with a characteristic knot structure, via solution-phase coupling of the LCP deprived of protection groups. Results of both the atmospheric and high pressure cyclization of two discussed peptides are presented in Figs 6 and 7.

Figs 6 and 7 show no difference between the two chromatograms, meaning that there is no additional structure of peptide formed in the sample, under high-pressure conditions. The same results were obtained for the sungsanpin analogue. In summary, we found that the application of high pressure did not cause formation of the lasso topology, and that the sole form obtained under these conditions was a branched-cyclic peptide.

### Cyclization of the linear core peptides of sungsanpin and chaxapeptin via tandem acyl shift

**Fig 8:**
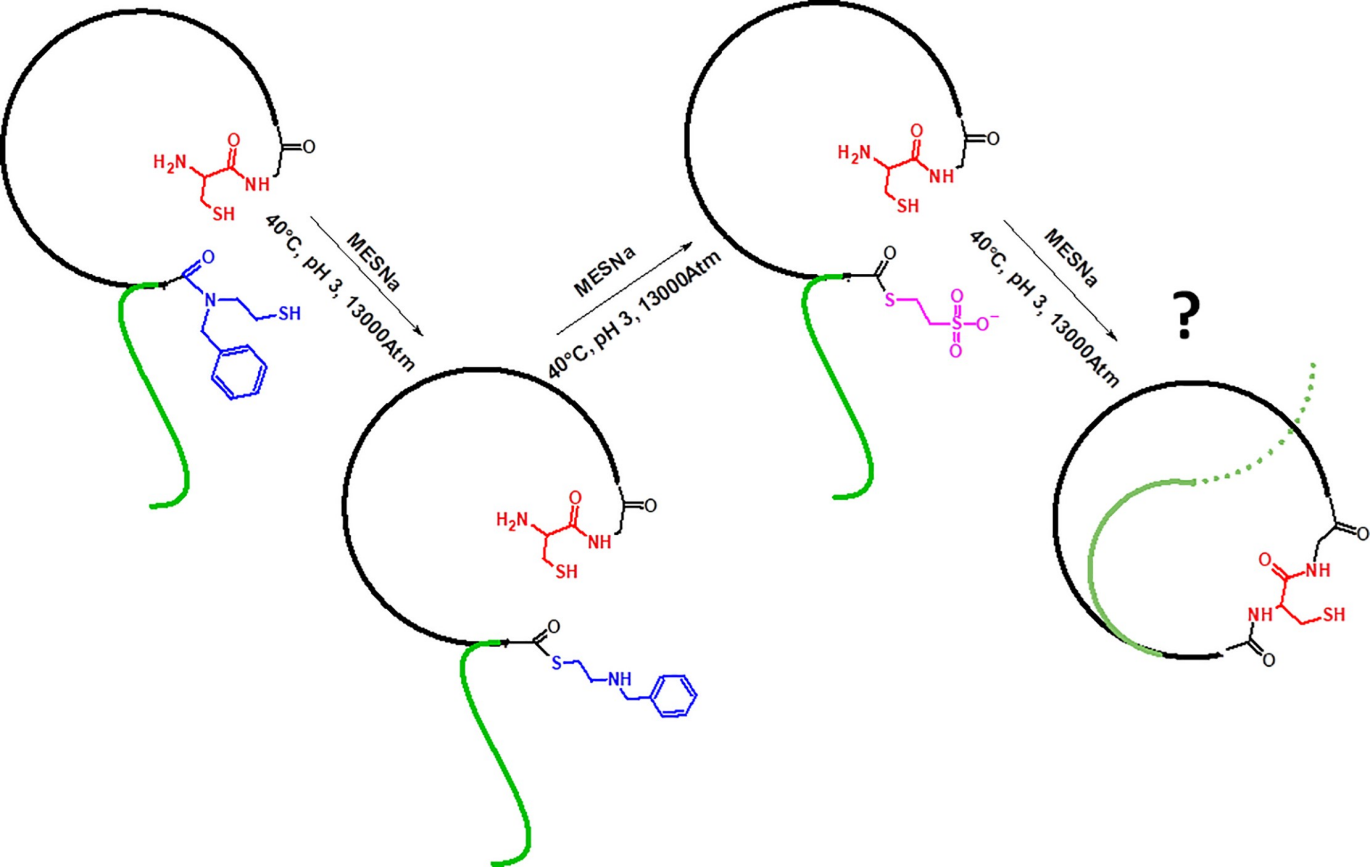
Schematic representation of tandem acyl shift approach.

We also tested a similar approach like in the previous section, though not based on the ordinary peptide bond coupling, but rather, on the tandem acyl shift ligation. Thus, reactions under high pressure were also studied, but by using conditions that required longer reaction times and were performed in an aqueous solution (as opposed to organic solutions). So, the fundamental differences, in comparison with the previous experiment, are the application of water as a solvent, and the elongation reaction time. We used a chemical ligation based on an N → S acyl shift, followed by ligation with the C-terminal cysteine residue and the S → N migration of acyl group. For this purpose, we designed the sungsanpin and chaxapeptin analogues containing an N-terminal cysteine residue instead of glycine. Moreover, we attached a 2-(ethylamine) ethane thiol moiety to the carboxyl group of the aspartic acid side chain as a thioester surrogate. According to literature data, thioethylalkilamido (TEA) thioesters subjected to acidic solutions have an N → S acyl shift, leading to formation of a thioester. This intermediate, in turn, reacted with an excess of other thiols (e.g. sodium mercaptoethanesulphonate-MESNa) present in the mixture (transthioesterification), providing a more reactive thioester. The scheme is presented in Fig 8.

**Fig 9:**
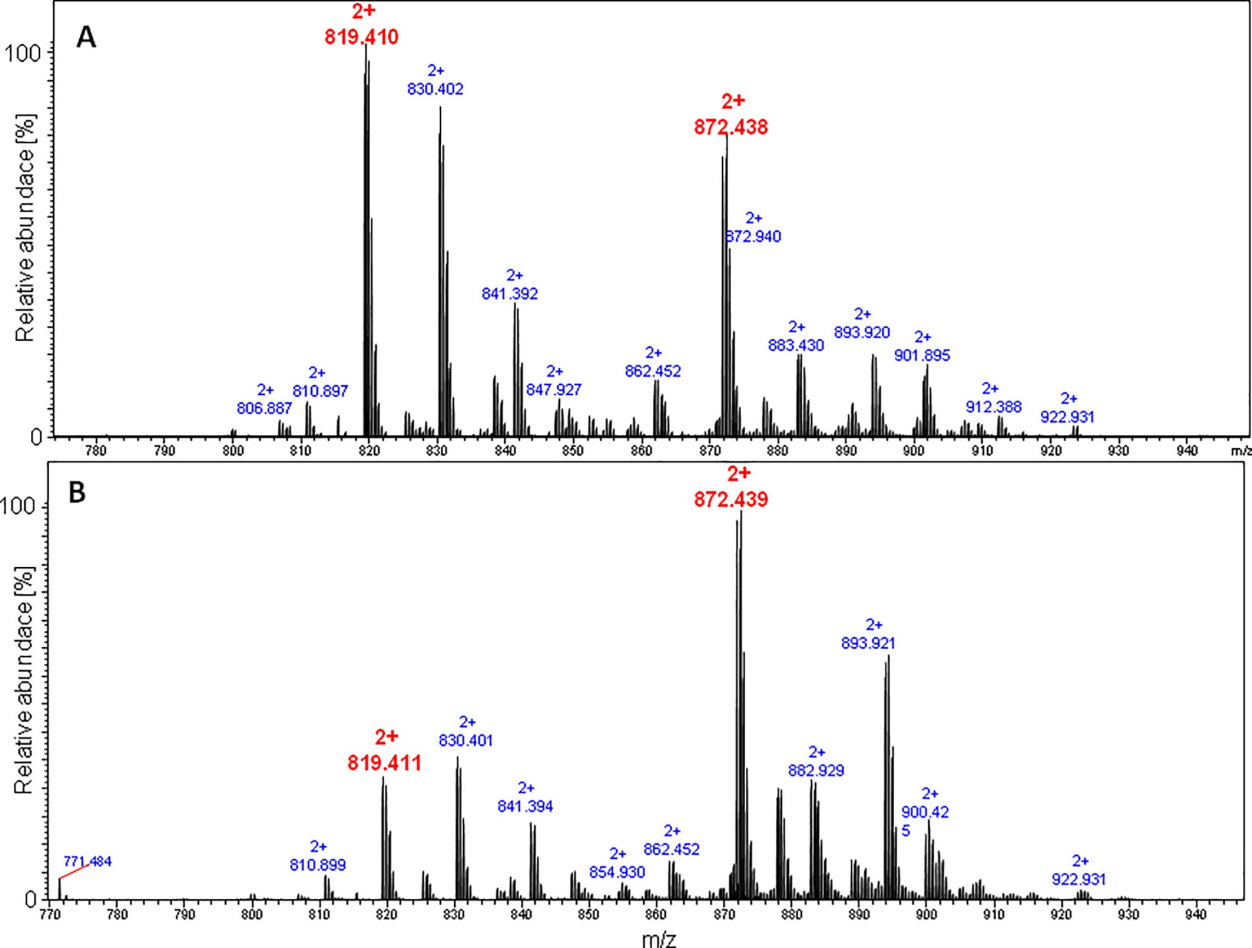
ESI-MS spectrum of the sungsanpin-1-Cys branched-cyclic analogue cyclized via tandem acyl shift over 24h and 48h.

**Fig 10:**
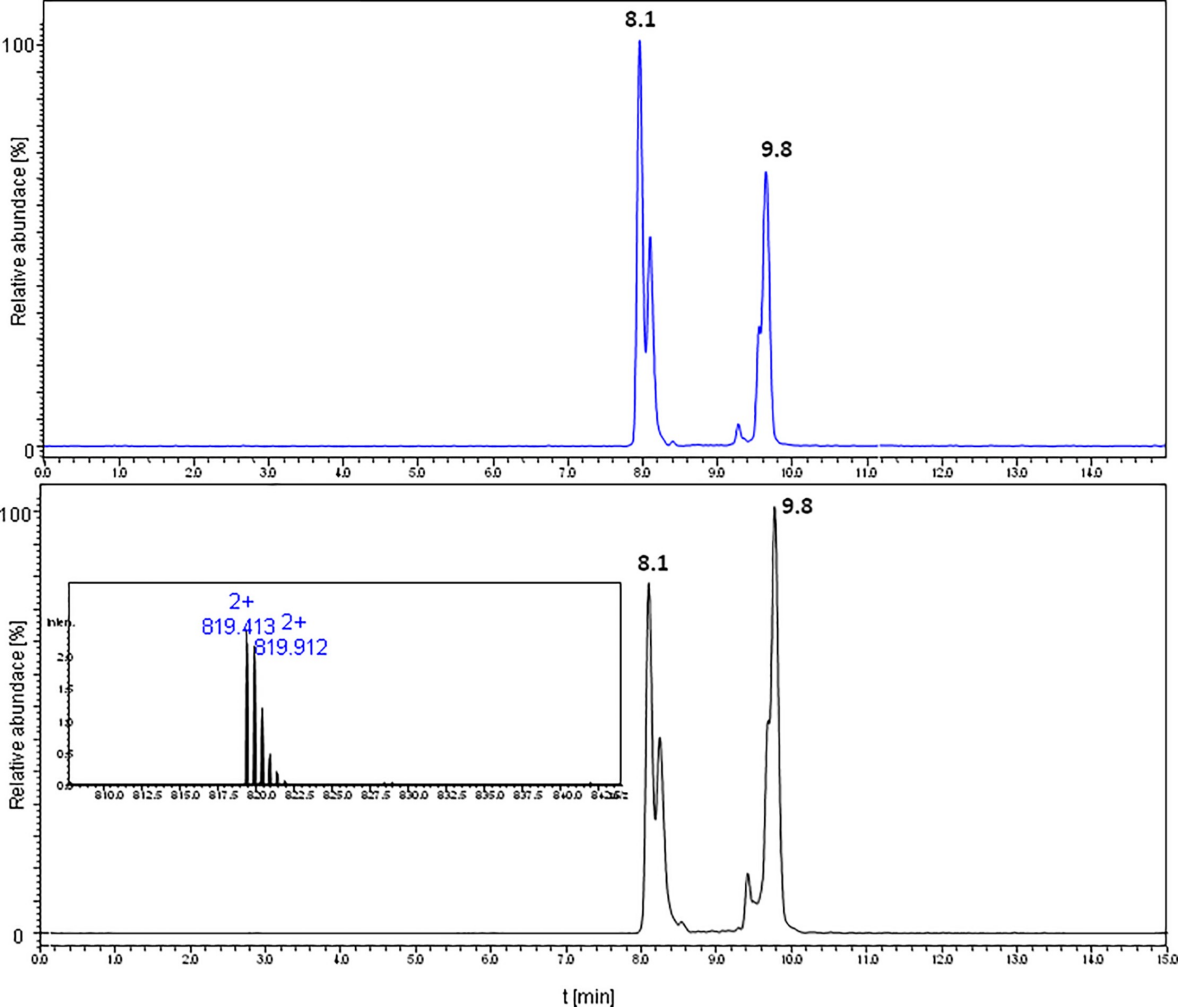
LC-MS chromatogram (XIC) of mixture obtained for peptides cyclized *via* tandem acyl shift of the sungsanpin-1-Cys LCP analogue (column thermostated in 60°C), under ambient (upper) and high pressure (bottom chromatogram).

We assembled the LCPs of sungsanpin- and chaxapeptin-1-Cys-containing analogues on a solid support, using a Chemmatrix Rink amide resin. After removal of the Asp side chain protection under very mild conditions (1%TFA/DCM), we attached an S-trityl-2-(ethylamino)ethanethiol (TEE) moiety by ordinary amide coupling using PyBOP. The assembled peptides were cleaved from resin, using a mixture of TFA:TIS:EDT:H_2_O (92.5: 2.5: 2.5: 2.5). LC chromatograms of purified LCPs are presented in S11 and S12 Figs of S1 Data. First, we tested the cyclization between the TEE-containing Asp residue and the N-terminal Cys. The reaction was carried out in a citric acid buffer (pH 3), and in the presence of MESNa over a 24h time period. The reaction was quenched by desalting on an OMIX^®^ C4 zip tip; this allowed elimination of the buffer and an excess amount of MESNa. Results of the cyclization reaction for the sungsanpin LCP analogue are shown in Fig 9. We observed a signal characteristic of a cyclic structure, even though a quite abundant peak from the linear peptide was still present in the MS spectrum. The prolonged incubation of samples, up to 48h, resulted in an improved yield of cyclic product. Similarly, the same experiment was performed under high pressure, using a piston apparatus; however, for practical reasons (e.g. limited stability of pressure over long time), the reactions were carried out only for 24h. This allowed for a qualitative view into the products, despite shortened reaction times and a lower yield of cyclic peptides. Afterwards, the mixture was analyzed by LC-MS (see Fig 10).

**Fig 11:**
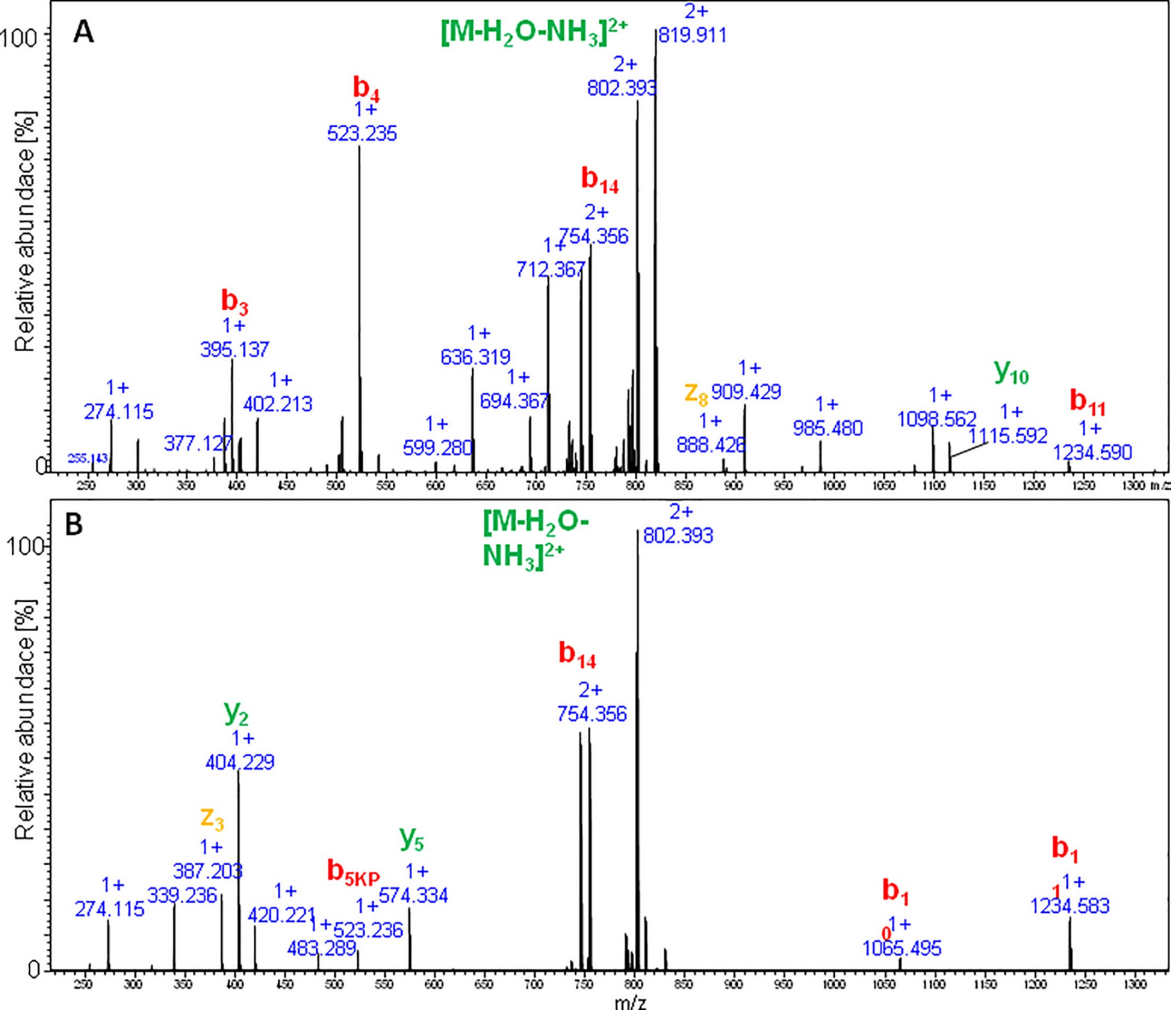
LC-MS/MS spectrum obtained for sungsanpin-1-Cys branched-cyclic analogue cyclized via tandem acyl shift. The upper spectrum at 8.1 min, the bottom at 9.8 min.

The comparison of LC-MS chromatograms obtained for both the atmospheric and high pressure cyclization of sungsanpin showed the presence of two signals corresponding to *m/z* 819 and *m/z* 413. Initially, the chromatographic peak at 9.8 min was broadened, but elevating the temperature during the LC separation to 60°C, resulted in a clearly narrower peak. The peak broadening is characteristic for cyclic peptides due to a conformational balance, especially at lower temperatures. Thus, at higher temperatures, the averaged peak was observed. Also, in this case, the appearance of an additional signal was intriguing, although, based on the results from previous experiments, the aspartimide formation was taken first into account. The analysis of the MS/MS spectrum at 8.1 min (see Fig 11) revealed the presence of a *z*_*8*_ ion, characteristic of an aspartimide-containing fragment. Moreover, there is a typical series of *b* ion types. These observations showed that the peptide eluting at 8.1 min exhibited a linear structure. The second signal at 9.8 corresponded to the branched-cyclic peptide. In the MS/MS spectrum, there are no occurrences of the *b* ion series (with the exception of the b_5KP_ ion) which include macrolactam ring fragments. Most fragments involve either the C-terminal tail or *b* fragments containing the whole cyclic part of the molecule. Besides these structures, no additional form of peptide was observed, even for high-pressure assembled peptides. An analogous outcome was achieved for the chaxapeptin-1-Cys LCP analogue. Thus, the conclusion was clear: these reaction conditions do not enable the formation of the characteristic lasso topology. Instead, a branched-cyclic peptide was formed.

### Solution-phase cyclization of the linear core peptide of the chaxapeptin-3,13-Cys analogue with a stiffened conformation because of a disulfide bridge

**Fig 12:**
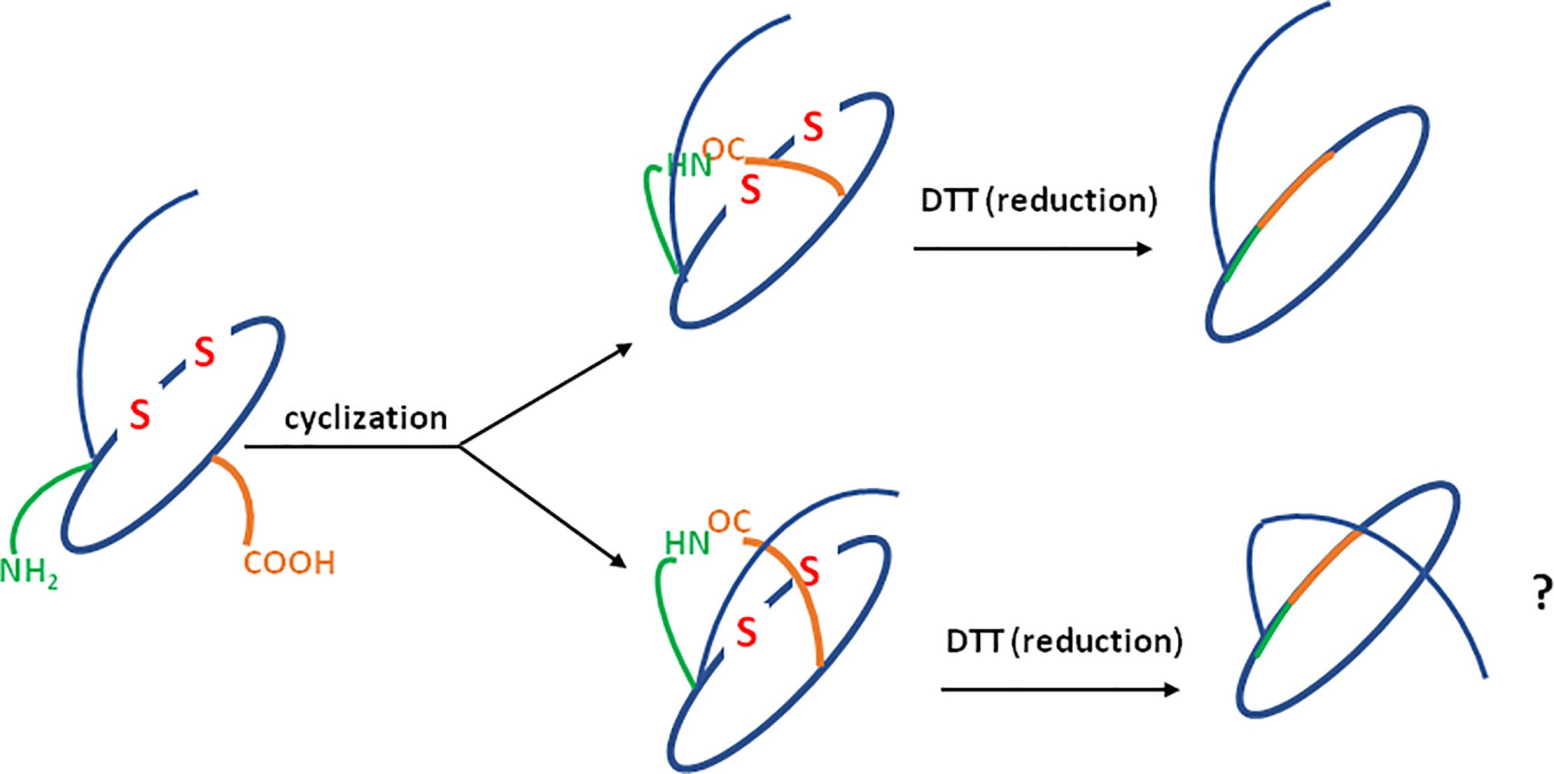
Scheme presenting the synthetic approach.

A further attempt to chemically synthesize lasso peptides was made by assembling a macrolactam ring through conventional peptide-bond coupling; the cyclization was carried out after the prior formation of a disulfide bond between two cysteine residues in positions 3 and 13. Additionally, the peptide had a protected ε-amino group of the lysine (benzyloxycarbonyl group) in order to avoid mixed coupling. In fact, we wanted to test the possibility of formation of the lasso motif upon prior stiffening of the tail. This idea relied on the synthesis of the LCP of a lasso peptide (on-resin) containing a disulfide bridge. The coupling between the α-amino group and the side chain carboxyl group was possible, in our opinion, from two sites of the protruding tail (Fig 12). The subsequent reduction of the disulfide bridge by dithiothreitol (DTT) could result in a mixture of both the branched- cyclic peptide and the lasso peptide.

**Fig 13:**
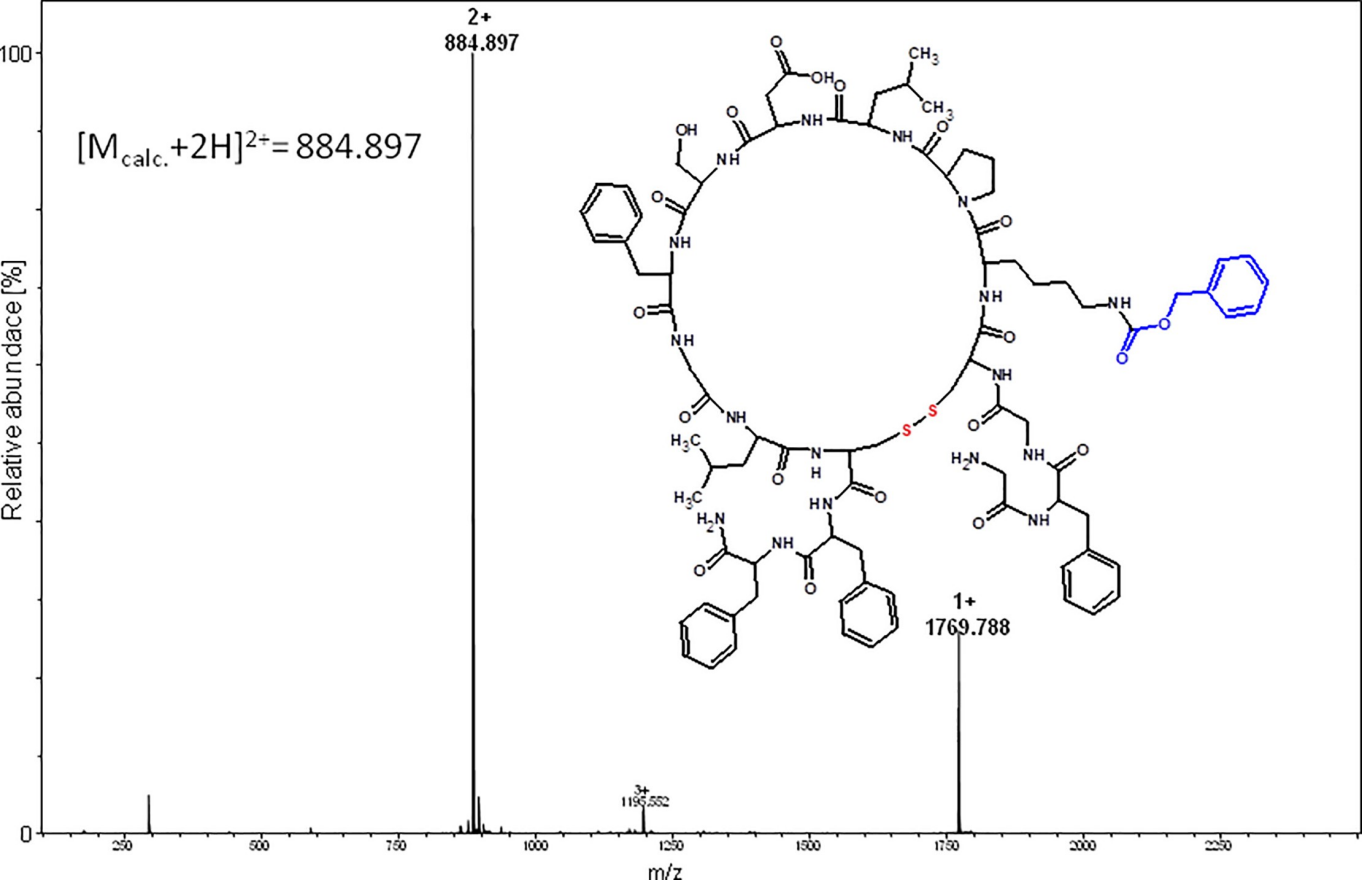
ESI-MS spectrum acquired for the oxidized chaxapeptin-3, 13-Cys branched-cyclic and oxidized analogue containing the benzyloxycarbonyl protecting group (purified product).

**Fig 14:**
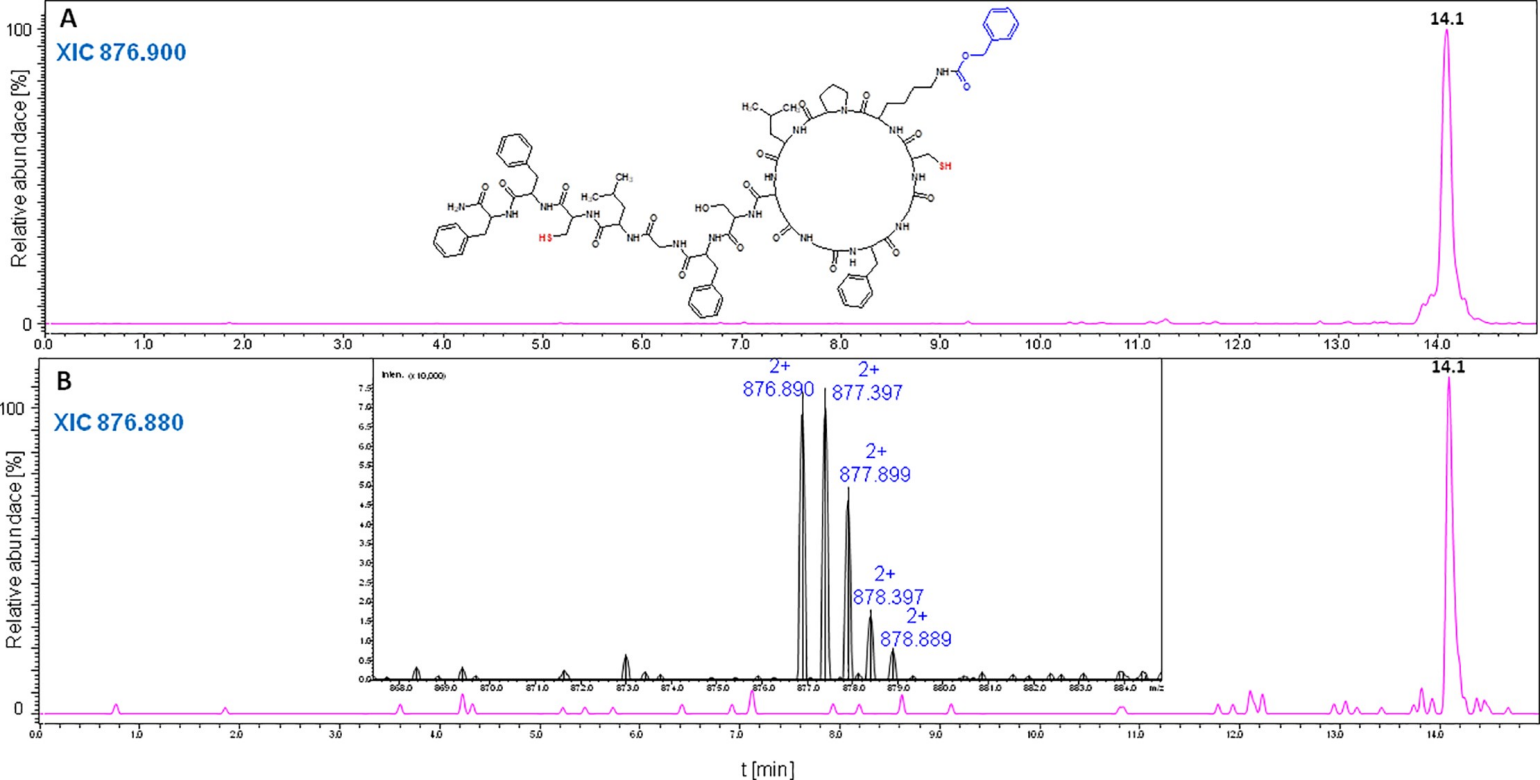
LC-MS chromatogram (XIC) acquired after cyclization of the oxidized chaxapeptin-3,13-Cys LCP analogue containing the benzyloxycarbonyl protecting group, under ambient pressure (A), and under high pressure (B). The column was thermostated in 55°C.

The on-resin formation of the disulfide bridge was selected (over the in-solution option) because it preferentially formed the intramolecular S-S bond. The ordinary oxidation with 5% DMSO/H_2_O, over 24h, was not sufficient; therefore, we used additional iodine. This yielded almost quantitative oxidation of the sulfhydryl groups within 2h. Fig 13 shows the ESI-MS spectrum of the purified and oxidized product. The oxidized peptide was subjected to cyclization (in solution-phase) using both ambient and high pressure in DMF. In order to eliminate the reducing agent and the buffer, the sample was desalted using OMIX^®^ C4 pipette tips, and was subsequently analysed by LC-MS. Results are presented in Fig 14. The chromatogram showed only one signal for both the ambient and high-pressure cyclized products. The separation was carried out at 55°C, because, a wide signal (corresponding to cyclic peptide) was observed at ambient temperature. The obtained data suggest the formation of a branched-cyclic peptide, but they do not suggest the formation of the desired lasso peptide.

## Conclusions

The data reported in literature suggest that, in many cases, the application of high pressure facilitates the formation of mechanically interlocked structures like rotaxanes [25]. Similar results were also obtained for [2] pseudorotaxanes based on two ether crowns, and secondary ammonium ion [26]. Because lasso peptides have [1] rotaxane like topology, we decided to test whether high pressure fostered the cyclization of a LCP into a threaded lasso peptide. To do so, we performed the cyclization both on a solid support (Chemmatrix^®^ resin), and in solution. The solution-phase synthesis was performed following the standard method, and using a tandem acyl- shift approach. We also tested the method involving the formation of a lactam bond in a LCP stiffened by a disulfide bridge. Those reactions were performed under atmospheric pressure, and under elevated pressure (13000 Bar). Our study revealed that the application of high pressure and the use of various solvents did not result in the formation of detectable quantities of lasso peptides. We also observed that the cyclization process did not depend on the type of solvent applied. The isomeric product formed in some of the reactions is, according to MS2 studies, a linear peptide, and the loss of water is the result of aspartimide formation. These results confirmed literature data that the cyclization of LCPs of lasso peptides results in the formation of branched-cyclic peptides. Moreover, the large scale purification of these peptides, using high-pressure liquid chromatography, appeared doable. In contrast to other examples of mechanically interlocked systems, lasso peptides do not appear to be sensitive to the conditions under which the cyclization process is carried out; these include the applied pressure and the type of solvent used in the reaction. Threaded lasso peptide formation in nature requires an ATP-dependent lactam synthetase [38,39]. Therefore, the biosynthesis of these peptides requires an energy source to mediate the core peptide pre-folding. This may explain why only using physical factors, such as the external pressure or solvent is not efficient in synthesizing the native lasso peptides. Moreover, according to IMS data, the differences in collision cross-sections of the lasso peptides, and branched cyclic peptides are small, especially in low charge states. Therefore, the reaction volume (ΔV_rxn_) is not sufficient to shift equilibrium significantly toward the lasso peptide at high-pressure conditions.

## Experimental

### Reagents

Solvents for peptide synthesis (analytical grade) were obtained from Sigma-Aldrich (dimethylformamide-DMF; trifluoroacetic acid-TFA) and J. T. Baker (diethyl ether). The (benzotriazol-1-yloxy)tripyrrolidinophosphoniumhexafluorophosphate (PyBOP) and (7-azabenzotriazol-1-yloxy)trispyrrolidinophosphoniumhexafluorophosphate(PyAOP) coupling reagent were obtained from Navoabiochem^®^. The H-Rink amide Chemmatrix^®^ resin was purchased from Sigma-Aldrich. All standard amino-acid derivatives were purchased from Peptydy.pl. Fmoc-Asp(O-PhiP)-OH was purchased from Novabiochem^®^, and Fmoc-Lys(Boc)-OH-^13^C_6_,^15^N_2_ was obtained from CortecNet (USA). Solvents for LC-MS and MS measurements: acetonitrile (MeCN), methanol (MeOH),formic acid (HCOOH) were purchased from Sigma Aldrich.

### Peptide preparation

The LCP of sungsanpin and chaxapeptin and their analogues were prepared on a solid support, according to the standard Fmoc protocol [40]. The coupling of respective amino acids residues was carried out using a PyBOP in DMF. Both the amino acid derivatives and the coupling agent were used in 3-fold excess. Additionally, the coupling reaction and Fmoc deprotection (25% piperydine/DMF) were supported by sonication over 15 min and 3 min, respectively [41]. During the synthesis, the Fmoc-Asp(O-2-PhiPr)-OH was used, together with the alternative side chain protection. This allowed on-resin de-protection of the Asp side chain, under mild conditions (1% TFA/DCM—20 x 2 min). Additionally, for the synthesis of the LCPs of the lasso peptides (cyclized in solution), a Fmoc-Lys(Z)-OH derivative was used. Z-protecting group remains intact after the TFA-based peptide cleavage from resin. This choice was dictated by two reasons: First, this alternative protecting group prevents the side reaction during solution-phase cyclization. Second, simplicity of the eventual removal of the protecting group by catalytic hydrogenolysis. Finally, a cyclization between the N-terminal glycine residue and the carboxyl group of the aspartic acid was performed, both in the solution- and solid-phase, using PyAOP (10-fold excess). The LCPs of the lasso peptides, cyclized via tandem acyl shift, contained S-trityl-2-(ethylamino)ethanethiol (TEE) on the Asp side chain carboxyl group, which was attached similarly to the amino acid residues, using a 3-fold excess of TEE and coupling agent. This derivative was attached to the Asp side chain upon prior removal of the labile protecting group (1% TFA/DCM). Chaxapeptine-3,13-Cys analogue, which was synthesized according to the aforementioned standard procedure, was oxidized on resin, using iodine in methanol (20 min). This approach favored the formation of the intramolecular disulfide bridge. The crude product was cleaved from the resin using a mixture of TFA/H_2_O/TIS (95:2.5:2.5, v/v) for 2h at room temperature. In the case of the Cys-containing peptide, 2.5% of 1,2-ethanedithiol (EDT) was also applied. The obtained peptides were precipitated in cold diethyl ether, and subsequently lyophilized.

### Synthesis of S-tritylcysteamine

5.68 g of cysteamine hydrochloride (50 mmol) was dissolved in 50 ml of DMF, in an Erlenmeyer flask equipped with a magnetic stirrer; 13.94 g of trityl hydrochloride was then added to the solution. The mixture was stirred at room temperature for 3 hours. The resulting solution was concentrated *in vacuo*, then neutralized with KOH/MeOH to a pH of 9, and concentrated *in vacuo* overnight. The resulting mixture was extracted four times with DCM (4x25 ml); all fractions were subsequently collected and washed with water (2x25 ml), brine (2x25 ml), dried over MgSO_4_, and finally evaporated on a rotavapor. The final product was crystallized from EtOAc by adding small portions of hexane. Crystals were left for growth overnight, at 4°C. Yield: 8.072 g (50.5%) of yellowish powder; TLC: 5% MeOH/CHCl_3_, Rf = 0.29, mp = 92–94°C, ESI-MS (*m/z*) cal. for [C_21_H_21_NS+NH]^+^ 320.146, found 320.144 (S7 Fig of S1 Data); ^1^H NMR (500 MHz, MeOD) δ 7.44 (dt, *J* = 8.6, 2.4 Hz, 6H), 7.31 (t, *J* = 7.6 Hz, 6H), 7.24 (t, *J* = 7.3 Hz, 3H), 2.46 (t, *J* = 7.0 Hz, 2H), 2.38 (t, *J* = 7.3 Hz, 2H) (S8 Fig of S1 Data).

### Synthesis of S-trityl-2-(ethylamino)ethanethiol (TEE)

*S*-Trt-cysteamine (250 mg, 7.82 x 10^-4^mol) was placed in a round bottom flask and dissolved in 15 ml of THF. After addition of acetaldehyde (44 μl, 7.82 x 10^-4^mol), the mixture was refluxed for over 1h. Sodium borohydride (118 mg) was then added to the solution in order to reduce the Schiff base, after which additional refluxing was performed, for 1h. Finally, 5 ml of water containing a few drops of acetic acid was added. This resulted in the decomposition of NaBH_4_. To remove the large amount of salt from the crude product, the mixture was placed in a separatory funnel and washed with a solution of sodium bicarbonate (3 x 5 ml), and subsequently with brine (2 x 5 ml). The organic layer was then dried with MgSO_4_, and evaporated under reduced pressure using a rotatory evaporator. Yield: 160 mg (60%) ESI-MS (*m/z*) cal. for [C_23_H_25_NS+H]^+^ 348.178, found 348.175 (S9 Fig of S1 Data); HPLC: Rt 10.5 min (S10 Fig of S1 Data)

### Cyclization of peptides by via tandem acyl shift

0.5 mg of TEE-containing peptide was dissolved in 500 μl of citric acid buffer (100 mM, pH 3), then 50 equivalents of MESNa was added. The resulting mixture was heated to 40°C over 24h. The reaction conditions were adopted from the procedure described by Taichi and Co. [33] Afterwards, the sample was desalted using OMIX^®^ C4 tips in order to remove the excess of MESNa. The peptide was eluted by 70% ACN in H_2_O; after dilution with water, it was analyzed by LC-MS.

### High pressure cyclization of peptides

Cyclization of the LCPs of sungsanpin and chaxapeptin was performed on solid support. For this purpose, 5 mg of resin containing an LCP of peptide was placed in clipped syringe with polypropylene filter. Then, 400 μl of DMF containing PyAOP (10-fold excess) and DIEA was added. The syringe was placed in the piston apparatus in the presence of silicon oil, and subjected to high pressure (13 000 Atm) for 3h. Solution-phase cyclization of the LCPs of sungsanpin and chaxapeptin was performed similarly, using the same reaction conditions (including pressure) as those described for the on-resin coupling. The cyclization of the LCPs of sungsanpin and chaxapeptin analogues were carried out via tandem acyl shift, according to the procedure described in section 2.8, using a syringe similarly introduced into the piston apparatus and subjected to high pressure over 24h.

### Mass spectrometric analysis

Mass spectrometry measurements were carried out on an Apex Ultra FT-ICR (Bruker, Germany) equipped with an electrospray source (ESI) ion funnel, and analyzed in the positive- ion mode. Before the run, the mass spectrometer was calibrated with a Tunemix mixture (Bruker Daltonics), following a quadratic method. Collision energy (10–20 eV; argon as a collision gas) was optimized during the MS/MS experiments for optimal fragmentation (the voltage over the hexapole collision cell varied from 15 to 30 V). An acetonitrile/water/formic acid (50:50:0.1) mixture was used as solvent for recording the mass spectra. The potential between the spray needle and the orifice was set to 4.5 kV.

### LC-MS

The LC-MS analysis of the peptides was performed on a Shimadzu LCMS-8050 equipped with a triple quadrupole mass spectrometer (using a UHPLC Nexera X2 system) and on an Agilent 6470 Triple Quadrupole LC/MS System equipped with a standard Jet Stream ESI source and Agilent Technologies 1290 Infinity II system. The LC-MS analysis on both instruments were carried out in SIM (selected ion monitoring) mode and with a Q1Q3 scan. The LC system was operated with a mobile phase consisting of solvent A: 0.1% formic acid in H_2_O and solvent B: 0.1% formic acid in MeCN. The gradient conditions (B %) were from 5 to 80% B, within 15 min. The flow rate was 0.2 mL/min, and the injection volume 5 μL. The separation was performed on an Aeris Peptide XB-C18 column (50 mm × 2.1 mm) with a 3.6 μm bead diameter. The peptide samples were dissolved in 400 μl of a water: acetonitrile mixture (80: 20). Most analysis were carried out on a Shimadzu IT-TOF, which is a hybrid system consisting of an ion trap and a time-of-flight mass analyzer; it also includes an electrospray (ESI) ion source. In our experiments, we set the potential between the spray needle and the orifice to 4.5 kV. The LC separation on this instrument was performed in the same condition as described above.

### NMR analysis

2.0 mg of a peptide were dissolved in 500 μL of a mixture of 10% D_2_O and 90% of H_2_O (v/v). After the peptide dissolved, the pH was not adjusted. In order to assign all cross-peaks from the Cα region the sample of sungsanpin with the same concentration in 100% of D_2_O was also prepared. All NMR experiments were performed on the 700 MHz (Avance III, Bruker) NMR spectrometer at 25°C. All NMR data were processed by NMRPipe and analyzed using a Sparky software [42, 43]. Complete assignment of the ^1^H and ^13^C resonances, for all peptides (S1 Table of S1 Data), was done by application of a standard and well-established procedures based on the inspection of the 2D-homonuclear TOCSY (with mixing times 10 and 80 ms) and ROESY (with mixing times 300) experiments [44].

### Purification and characterization of peptides

After release from the resin, the crude peptide products were analyzed using a Thermo Separation HPLC system with UV detection (210 nm) on a Vydac Protein RP C18 column (4.6 × 250 mm, 5 μm), with a gradient elution of 0%–80% S2 in S1 (S1 = 0.1% aqueous TFA; S2 = 80% acetonitrile + 0.1%) for 40 min (flow rate 1 mL/min). TEE was analyzed on an Aeris Peptide XB-C18 column (50 mm × 2.1 mm; 3.6 μm bead diameter) using a Shimadzu IT-TOF instrument equipped with a PDA detector. The separation conditions were similar to those described in section 2.3. The main carbonylated product was purified using a preparative reversed-phase HPLC on a Vydac C18 column (22 mm x 9 250 mm), using solvent systems: S1 0.1% aqueous TFA, S2 80% acetonitrile + 0.1% TFA, linear gradient from 50 to 100% of S2 for 55 min, flow rate 7.0 ml/min, UV detection at 210 and 280 nm. The resulting fractions were collected and subjected to lyophilisation. The identities of the products were confirmed by MS analysis, using an Apex Ultra FT-ICR (Bruker, Germany) mass spectrometer equipped with an electrospray (ESI) ionization source.

## Supporting information

S1 Data (DOCX)
