# Supplementary material for: Attempting to synthesize lasso peptides using high pressure
Source: PLoS One. 2020 Jun 24;15(6):e0234901. doi: 10.1371/journal.pone.0234901 (PMC7314030; doi:10.1371/journal.pone.0234901)
Supplement: S1 Data — (DOCX) [file pone.0234901.s001.docx]

**ATTEMPTING TO SYNTHESIZE LASSO PEPTIDES USING HIGH PRESSURE**

Mateusz Waliczek^a^, Magdalena Wierzbicka^a^, Maciej Arkuszewski^a^, Monika Kijewska^a^, Łukasz Jaremko^b^, Priyadharshni Rajagopal^b^, Kacper Szczepski^a,b^ ,
Amanda Sroczyńska^a^, Mariusz Jaremko^b^*, Piotr Stefanowicz^a^ *

*^a^ Faculty of Chemistry, University of Wrocław, F. Joliot-Curie 14, 50-383 Wroclaw, Poland*

*^b^* *King Abdullah University of Science and Technology (KAUST), Biological and Environmental Sciences & Engineering Division (BESE), Thuwal, 23955-6900, Saudi Arabia.*

**Table. S1** NMR data for H-Gly-Phe-Gly-Ser-Lys-Pro-Ile-Asp-Ser-Phe-Gly-Leu-Ser-Trp-Leu-NH_2_ [ppm]

| **Res.** | **C^α^** | **C^β^** | **C^γ^** | **C^δ^** | **C^ε^** | **C^z^** | **H^α^** | **H^β^** | **H^γ^** | **H^δ^** | **H^ε^** | **HN** | **H^z^** |
| --- | --- | --- | --- | --- | --- | --- | --- | --- | --- | --- | --- | --- | --- |
| **Gly1** | 48.891 | - | - | - | - | - | 3.859, 3.767 | - | - | - | - | 8.430 | - |
| **Phe2** | 59.114 | 40.254 | - | 129.101 | 128.904 | 127.320 | 4.213 | 3.169, 2.851 | - | 7.208 | 7.287 | 8.444 | - |
| **Gly3** | 45.517 | - | - | - | - | - | 3.871 | - | - | - | - | 8.485 | - |
| **Ser4** | 58.473 | 64.877 | - | - | - | - | 4.480 | 3.752 | - | - | - | 8 | - |
| **Lys5** | 58.107 | 32.441 | 27.148 | 28.918 | 46.446 | - | 4.310 | 2.174 | 1.224 | 1.450 | 3.331 | 7.898 | - |
| **Pro6** | 63.348 | 33.051 | 27.313 | 50.075 | - | - | 4.349 | 1.721 | 1.510 | 3.281 | - | - | - |
| **Ile7** | 61.451 | 39.358 | 32.503, 17.915 | 13.416 | - | - | 4.061 | 1.719 | 1.779, 0.739 | 0.777 | - | 8.123 | - |
| **Asp8** | 55.561 | 39.958 | - | - | - | - | 4.221 | 3.038 | - | - | - | 7.931 | - |
| **Ser9** | 58.214 | 63.813 | - | - | - | - | 4.617 | 3.738 | - | - | - | 8.134 | - |
| **Phe10** | 59.026 | 42.112 | - | 129.047 | 128.751 | 127.220 | 4.281 | 2.793, 2.610 | - | 7.105 | 7.237 | 8.726 | 7.198 |
| **Gly11** | 51.054 | - | - | - | - | - | 3.729, 3.520 | - | - | - | - | 8.035 | - |
| **Leu12** | 54.638 | 42.575 | 27.785 | 23.649, 23.556 | - | - | 4.521 | 1.507 | 1.902 | 0.786, 0.665 | - | 8.528 | - |
| **Ser13** | 58.449 | 64.245 | - | - | - | - | 4.385 | 3.757 | - | - | - | - | - |

| **Trp14^ᶿ^** | 55.183 | 29.641 | | - | 124.558 | 119.554 | | 112.115, 118.257 | | 4.075 | | | 3.209 | - | 7.123 | 7.054 | | 8.230 | | 7.388, 7.523 | |
| --- | --- | --- | --- | --- | --- | --- | --- | --- | --- | --- | --- | --- | --- | --- | --- | --- | --- | --- | --- | --- | --- |
| **Leu15** | 53.111 | 42.766 | 29.456 | | 25.340, 25.394 | | - | | - | | 4.648 | 1.371 | | 1.586 | 0.831, 0.744 | | - | | 7.804 | | - |

**ᶿ**In Trp14 residue: the additionally signals C^H2^122.136 ppm, H^H2^ 7.138 ppm are present.

# The accuracy of chemical shift determination is 0.01 ppm for ^1^H and 0.1 ppm for ^13^C resonances.

* Stereospecifically assigned H^β^ protons first and second values correspond to H^β1^ and H^β2^, respectively.

$ ring carbons of Mim C^3^, C^4^  and C^5^ (up to down) are connected to the H^δ^, H^f^ and H^ε^ protons, respectively.

**^^^**For Val C^γ^ carbons first and second values correspond to C^γ1^ and C^γ2^, respectively, analogically with the H^γ^ protons.

**Fig S 1.** LC-MS chromatogram (XIC m/z 796.6) obtained for chaxapeptin LCP analogue (C-terminal amide group) cyclized in ambient and high pressure using mixture of ACN/THF.

**Fig S 2.** ESI-MS spectrum obtained for sungsanpin LCP analogue (C-terminal amide group).

**Fig S 3**. ESI-MS spectrum obtained for linear and isotopically labeled sungsanpin -^13^C_6_,^15^N_2_ LCP analogue

**Fig S 4**. LC-MS chromatogram (XIC m/z 816.410) obtained for chaxapeptin LCP analogue (C-terminal amide group).

**Fig S 5.** HPLC chromatogram of purified chaxapeptin-amide LCP analogue containing protected (benzyloxycarbonyl group) ε-amino group of lysine.

**Fig S 6.** HPLC chromatogram of purified sungsanpin-amide LCP analogue containing protected (benzyloxycarbonyl group) ε-amino group of lysine.

**Fig S 7.** ESI-MS spectrum obtained for S-Trt-cysteamine (calc. [M+H]^+^ 320.146). The abundant signal at m/z 243 was formed in the gas phase.

**Fig S 8.** NMR spectrum of S-Trt-cysteamine.

**Fig S 9**. ESI-MS spectrum obtained S-Trityl-2-(ethylamino)ethanethiol (TEE) (calc. [M+H]+ 348.178). The abundant signal at m/z 243 was formed in the gas phase.

**Fig S 10.** LC chromatogram of purified S-trityl-2-(ethylamino)ethanethiol (TEE) at 210 nm.

**Fig S 11.** LC chromatogram obtained for chaxapeptin LCP Cys analogue (C-terminal amide group) containing TEE on the Asp side chain at 210 nm (PDA detector).

**Fig S 12.** LC chromatogram obtained for sungsanpin LCP Cys-analogue (C-terminal amide group) containing TEE on the Asp side chain at 210 nm (PDA detector).

**Fig S 13**. LC-MS chromatogram (XIC m/z 816.410) obtained for sungsanpin branched-cyclic peptide (C-terminal amide group) containing protected ε-amino group of lysine (benzyloxycarbonyl group) cyclized in solution under ambient and high pressure in the mixture of (THF/ACN/DMF; 40:40:20).

**Fig S 14.** ESI-MS spectrum obtained for sungsanpin branched-cyclic peptide Cys-analogue (C-terminal amide group) via tandem acyl shift **A.** cyclization over 24h **B**. Cyclization over 48h

**Fig S 15.** LC-MS chromatogram (XIC) obtained for chaxapeptin branched-cyclic peptide Cys-analogue (C-terminal amide group) via tandem acyl shift**.** Cyclization over 24h

**Fig S 16.** ESI-MS/MS spectrum obtained for chaxapeptin branched-cyclic peptide Cys-analogue (C-terminal amide group) via tandem acyl shift**.** Cyclization over 24h. **A.** Fragmentation of signal at 8.2 min **B.** Fragmentation of signal at 10.1 min
